# Prevalence of obesity and overweight, its clinical markers and associated factors in a high risk South-Asian population

**DOI:** 10.1186/s40608-015-0044-6

**Published:** 2015-03-18

**Authors:** Faridah Amin, Syeda Sadia Fatima, Najmul Islam, Anwar H Gilani

**Affiliations:** Natural Product Research Unit, Department of Biological and Biomedical Sciences, The Aga Khan University Medical College, Karachi, 74800 Pakistan; Department of Biological and Biomedical Sciences, The Aga Khan University Medical College, Karachi, 74800 Pakistan; Department of Medicine, The Aga Khan University Medical College, Karachi, 74800 Pakistan; College of Health Sciences, Mekelle University, PO Box 1871, Mekelle, Ethiopia

**Keywords:** Obesity, Body mass index, Waist circumference, Waist-hip ratio, Body fat percentage

## Abstract

**Background:**

Obesity is a global epidemic, which is a risk factor for cardiovascular diseases and metabolic abnormalities. It is measured by body mass index (BMI), waist circumference (WC), waist-hip ratio (WHR), body fat (BF) distribution and abdominal fat mass, each having its own merits and limitations. Variability in body composition between ethnic groups in South-Asians is significant and may not be truly reflected by BMI alone, which may result in misclassification. This study therefore, aims to determine the frequency of obesity, body fat composition and distribution, in a high risk population of an urban slum of Karachi, Pakistan. This survey included 451 participants selected by systematic sampling who were administered pre-tested questionnaires on socio-demographics, diet and physical activity. Chi-square was used to determine the association between categorical variables and multiple linear regression was used for quantitative variables. A P value of less than 0.05 was considered significant.

**Results:**

Classified by BMI, 29% study subjects were overweight and 21% obese (58.7% with central obesity). Body fat percent (BF%) classified 81% as overweight. Females were more obese (P 0.03) with higher prevalence of central obesity (P <0.001) and WHR (P 0.003) but with a lower muscle mass (P 0.001). Activity score and muscle mass showed inverse linear association with BF% whereas, WC, weight, BMI and WHR had a positive linear association with BF%. The relationship between BMI and BF% was quadratic with a weaker association at lower BMI. Adjusting for socio-demographic variables, BF%, weight, diastolic blood pressure (DBP), BMI and score on the diet questionnaire had a positive linear association with WC, while WC, WHR and BP had a positive linear association with BF%. BF%, muscle content and WC had a positive linear association with BMI.

**Conclusion:**

Considering lower cut-offs for South-Asians BMI and WC, this study showed a high prevalence of obesity among a sub-urban population of Karachi, which was even higher when BF% was measured. Considering the rising prevalence of non-communicable diseases, BF%, WC, WHR and BMI measurements are convenient and feasible means of identifying population at risk and hence addressing it through public awareness and early detection.

**Electronic supplementary material:**

The online version of this article (doi:10.1186/s40608-015-0044-6) contains supplementary material, which is available to authorized users.

## Background

Obesity is a global epidemic and is an important risk factor for developing cardiovascular diseases (CVD), including diabetes, hypertension and dyslipidemia [1]. In developing countries, the rate of obesity has tripled, which has been attributed primarily to adopting a modern lifestyle with less physical activity and excessive consumption of energy dense foods [2].

Body mass index (BMI) recommended by the World Health Organization (WHO) to classify obesity, is the gold standard for identifying patients at risk of adverse health outcomes. Various epidemiological studies have shown a direct association between BMI and the risk of medical complications and mortality rate. WHO and the National Institute of Health have provided guidelines for classifying obesity based on BMI. The guidelines have proposed that adults who have a BMI ≥30 kg/m^2^are obese and are generally at higher risk for adverse health events than overweight (BMI between 25.0 and 29.9 kg/m^2^) or lean (BMI between 18.5 and 24.9 kg/m^2^) [3].

Besides BMI, another important risk factor for obesity related diseases is the body fat distribution. Expensive imaging techniques are required for the precise measurement of abdominal fat content and it is also known that waist circumference (WC) has a correlation with abdominal fat mass, therefore, WC is often used as a surrogate marker for measuring abdominal fat mass [3], even though it doesnot take into account variation in height. Central obesity and a higher waist-hip ratio (WHR) have been linked with the development hyperinsulinemia, insulin resistance, dyslipidemia, pro-inflammatory and pro-thrombotic clinical states [1]. Moreover, excess body fat has also been regarded as the single most important determinant of type 2 diabetes, whereas higher muscle mass is associated with better insulin sensitivity and lower risk of diabetes. [4,5].

The burden of obesity and obesity related diseases is particularly higher in the middle-income countries of Eastern Europe, Latin America and Asia, where obesity ranks just below underweight as the fifth most common cause of disease burden. This increased risk of cardio-metabolic diseases in Asians may be due to increased abdominal obesity [2,6]. It has also been shown that although in Europeans, a BMI of 30 kg/m^2^ correlates with about 25% body fat in males and 30% body fat in females, however, for the same age, sex, and BMI, South-Asians have an increased body fat percentage (BF%), both total and in the abdominal region, lesser lean mass, skeletal muscle and bone mineral content along with a higher risk for CVD [6-8]. The significant variability in body composition between ethnic groups may not be truly reflected by measuring only BMI or other markers as each has its own limitations [9]. Therefore in 2002, WHO recommended lower cut-off points of BMI (less than 18 · 5 kg/m^2^ underweight; 18 · 5–23 kg/m^2^ increased but acceptable risk; 23–27 · 5 kg/m^2^ increased risk; and 27 · 5 kg/m^2^ higher high risk) and normal WC (less than 80 cm for women and 90 cm for men) for high risk populations including South-Asians. Even with the low cut-off values, Asians show variations in the relationship among BMI, BF% and body fat distribution [10]. Therefore, using WC or BMI alone to classify individuals according to fatness may result in misclassification because of the varying contributions of body composition [10-12].

National Health Survey and studies in Pakistan show that while obesity and diabetes are more prevalent in urban dwellers [13,14], yet the prevalence is also high in rural areas [15]. In Metroville health study, 34% men and 49% women were found to be over-weight/obese, while increased WHR was observed in 41% and 72% of men and women respectively [16]. There is a general perception as if obesity or overweight is prevalent more in affluent societies but there is now emerging evidence that obesity is growing even in the poor population.

This study therefore, aims to determine the prevalence of obesity, body fat composition and distribution, in an urban slum (Hijrat Colony) in Karachi, Pakistan. Most of the residents belong to the labour class and many of them have living standards below the poverty line [17], therefore, it will be interesting to study the prevalence of obesity, its association with dietary patterns, physical activity and correlation of BMI with body fat composition in this population.

## Method

This study was approved by an institutional Ethical Review Committee of Aga Khan University (2512-BBS-ERC-13). It is a cross sectional study, conducted in Hijrat colony, Karachi an urban slum near Mai-kolachi bypass. There are approximately 4000 households in this area with a total population of more than 25,000 [18]. Assuming an obesity prevalence of 46% [19], a sample size of 451 was calculated with a confidence interval of 95% and a relative precision of 10%. Adults between the ages of 18–65 years who were residents of Hijrat colony and consented to participate were included. A written informed consent was obtained from the participants. Pregnant females, physically or mentally disabled, bed ridden patients, people suffering from a chronic ailment like malignancy and patients on steroid therapy for more than 2 weeks were excluded. Systematic sampling was done and every 10^th^ household was selected for participation. In case of non-consent the next house hold was visited. Only one available family member was selected for participation/household. After taking a written consent, a pre-tested questionnaire on socio-demographics, diet intake [20] and physical activity [21] was administered by the research officer. The total scores on the diet questionnaire (MEDFICTS) and international physical activity questionnaire (IPAQ) were calculated. Other parameters were measured as mentioned in operational definitions (See Additional file 1).

Data was entered and analysed on SPSS version 20. Mean and standard deviation was calculated for quantitative variables like age, body mass index, Blood pressure (BP), waist circumference and body composition. For categorical variables, frequency and proportion was calculated. Chi-square was used to determine the association between categorical variables and student’s t-test and linear regression was used for quantitative variables. A p-value of less than 0.05 was considered significant. Pearson (r) or Spearman’s (r_s_) correlation co-efficient was calculated to determine the strength of correlation for parametric and non-parametric variables respectively. Multiple linear regression was used to assess the association of BMI, WC, weight and body fat percent (BF%) with various independent variables. Goodness of fit was measured by co-efficient of determination (r^2^).

## Results

Among the participants, 54% (245) were males, 46% (206) were females and majority of the participants (86%) were married. Regarding ethnicity, 51% were Pushto, 25% were Punjabi, 12.4% were Urdu speaking while rest were from northern areas, Afghanistan or Baluchistan. More than half (60%) were illiterate and among the remaining 40%, 14% completed up to primary education and 22% attended secondary school while only 4% had completed their graduation.

Table 1 shows the mean clinical measures of enrolled male and female participants and their comparison. The weight (P <0.001), random blood glucose (P 0.02), BMI (P 0.008), hip circumference (P <0.001), waist-hip ratio (P 0.009), BF% (P 0.005) and muscle content (P 0.001) was significantly different among males and females.

**Table 1:** **Mean anthropometric measures of male and female participants**

**Parameter**	**Gender**	**Mean** **±** **SD**	**Total mean** ** ± SD**	**P value**
**Systolic BP (mmHg)**	Male	118.1 ± 22.0	118.6 ± 23.2	0.65
	Female	119.2 ± 24.7		
**Diastolic BP (mmHg)**	Male	70.2 ± 13.2	69.4 ± 13.1	0.12
	Female	68.3 ± 12.8		
**Waist circumference (cm)**	Male	87.0 ± 12.2	86.9 ± 12.0	0.78
	Female	86.7 ± 11.7		
**Weight (kg)**	Male	64.6 ± 15.0	61.1 ± 14.8	<0.001*
	Female	57.5 ± 14.6		
**Random blood glucose (mg/dl)**	Male	151.8 ± 84.5	144.5 ± 74.6	0.02*
	Female	135.8 ± 59.9		
**BMI (kg/m** ^**2**^ **)**	Male	22.7 ± 6.07	23.4 ± 5.5	0.008*
	Female	24.1 ± 6.33		
**Hip circumference (cm)**	Male	97.9 ± 9.58	99.9 ± 10.2	<0.001*
	Female	102.2 ± 10.3		
**Body fat (%)**	Male	31.5 ± 15.3	33.1 ± 12.6	0.005*
	Female	34.9 ± 7.98		
**Muscle content (%)**	Male	41.2 ± 16.6	37.2 ± 13.4	<0.001*
	Female	32.2 ± 4.07		
**Activity score (MET-min/wk)**	Male	2833.8 ± 3663.5	2607.9 ± 3190.1	0.09
	Female	2339.0 ± 2496.04		
**Waist-hip ratio**	Male	0.89 ± 0.12	0.87 ± 0.1	0.009*
	Female	0.84 ± 0.05		

*p value <0.05, differences determined by student’s t-test.

Table 2 shows the association of normal and abnormal clinical parameters with gender. Among participants, 59% did not report any co-morbid illnesses (such as diabetes, hypertension, hyperlipidemia and other chronic illnesses for more than 6 months) while 11% were diabetic and 17% were hypertensive (Table 2). Around two-fifth (39%) were on regular medications for various chronic diseases like diabetes, hypertension, heart diseases, dyslipidemia and musculoskeletal pains. Regarding family history of illnesses, 33.5% had a family history of diabetes, 41% hypertension, 17.3% hyperlipidemia and 10% had a family history of ischemic heart disease. Among participants, 37.3% reported major stressors and 69% of the males had stress more than 3 days a week as compared to 54% of the females, which was statistically significant (P 0.005). Among males, 12% smoked cigarettes and 21% males used chewable tobacco, while a small number of females (2%) used either tobacco or smoked cigarettes.

**Table 2:** **Distribution of different parameters among males and females**

	**Male (245) n (%)**	**Females (206) n (%)**	**Total (451) n (%)**	**P value**
**Co-morbid illnesses**				
None	144 (59)	123 (60)	267 (59.2)	
Diabetes	29 (11.8)	22 (10.6)	51 (11.3)	0.86
Hypertension	42 (17.1)	35 (16.9)	77 (17)	0.83
Dyslipidemia	7 (2.8)	3 (1.4)	10 (2.2)	0.42
**Addictions**				
None	118 (48.1)	196 (95.1)	314 (69.6)	
Yes	127 (51.9)	10 (4.8)	137 (30.3)	<0.001*
**BMI (kg/m** ^**2**^ **)**				
Normal (18.5-23)	77 (31.4)	58 (28.2)	135 (29.9)	
Underweight (<18.5)	48 (19.8)	41 (19.9)	89 (19.7)	0.67
Overweight (23.1-27.5)	85 (35.1)	48 (23.3)	133 (29.4)	0.23
Obese (>27.5)	34 (14)	60 (29.1)	94 (20.8)	0.002
**Systolic blood pressure (mmHg)**				
Normal <130	179 (73.1)	139 (67.8)	318 (70.5)	
≥130	66 (26.9)	67 (32.5)	133 (29.4)	0.22
**Diastolic blood pressure (mmHg)**				
Normal <85	211 (86.1)	182 (88.3)	393 (87.1)	
Abnormal ≥ 85	34 (13.9)	24 (11.9)	58 (12.9)	0.53
**Waist circumference (cm)**				
Normal	138 (56.3)	54 (26.2)	192 (82.5)	
Central obesity	107 (43.7)	152 (73.8)	259 (57.4)	<0.001*
**Waist hip ratio (cm)**				
Normal	71 (29)	87 (42.2)	158 (35)	
Abnormal	174 (71)	119 (57.8)	293 (65)	0.003*
**Body fat percent**				
Normal	40 (16.3)	34 (16.5)	74 (16.4)	
Underweight	3 (1.2)	8 (3.8)	11 (2.4)	0.11
Overweight	146 (59.5)	117 (56.7)	263 (58.3)	0.82
Significantly overweight	56 (22.8)	47 (22.8)	103 (22.8)	0.96
**Muscle Mass (%)**				
Normal	68 (27.7)	59 (28.6)	127 (28.1)	
Low	5 (2)	28 (13.6)	33 (7.3)	0.001*
High	150 (61.2)	141 (68.4)	291 (64.5)	0.70
**Random blood glucose (mg/dl)**				
Normal < 200	214 (87.3)	182 (88.3)	396 (87.8)	
Abnormal ≥ 200	31 (12.7)	24 (11.7)	55 (12.2)	0.73

*p value <0.05, associations determined by uni-variable logistic regression.

A total of 29% were overweight according to South-Asian cut-off (BMI 23.1-27.5 kg/m^2^) and 21% (BMI > 27.5 kg/m^2^) were found to be obese among the participants. 58.7% were centrally obese while 81% were overweight or significantly overweight classified through BF%. Females were more likely to be obese than males (P 0.03) and also more likely to have central obesity (P <0.001) and a higher waist-hip ratio (P 0.003) with a lower muscle mass (P 0.001) compared to males (Table 2).

**Figure 1:**
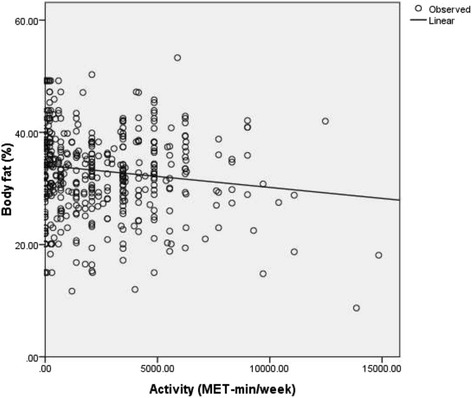
**Association of activity score with body fat percentage.** (P 0.03) (pearson correlation co-efficient r = −0.18).

**Figure 2:**
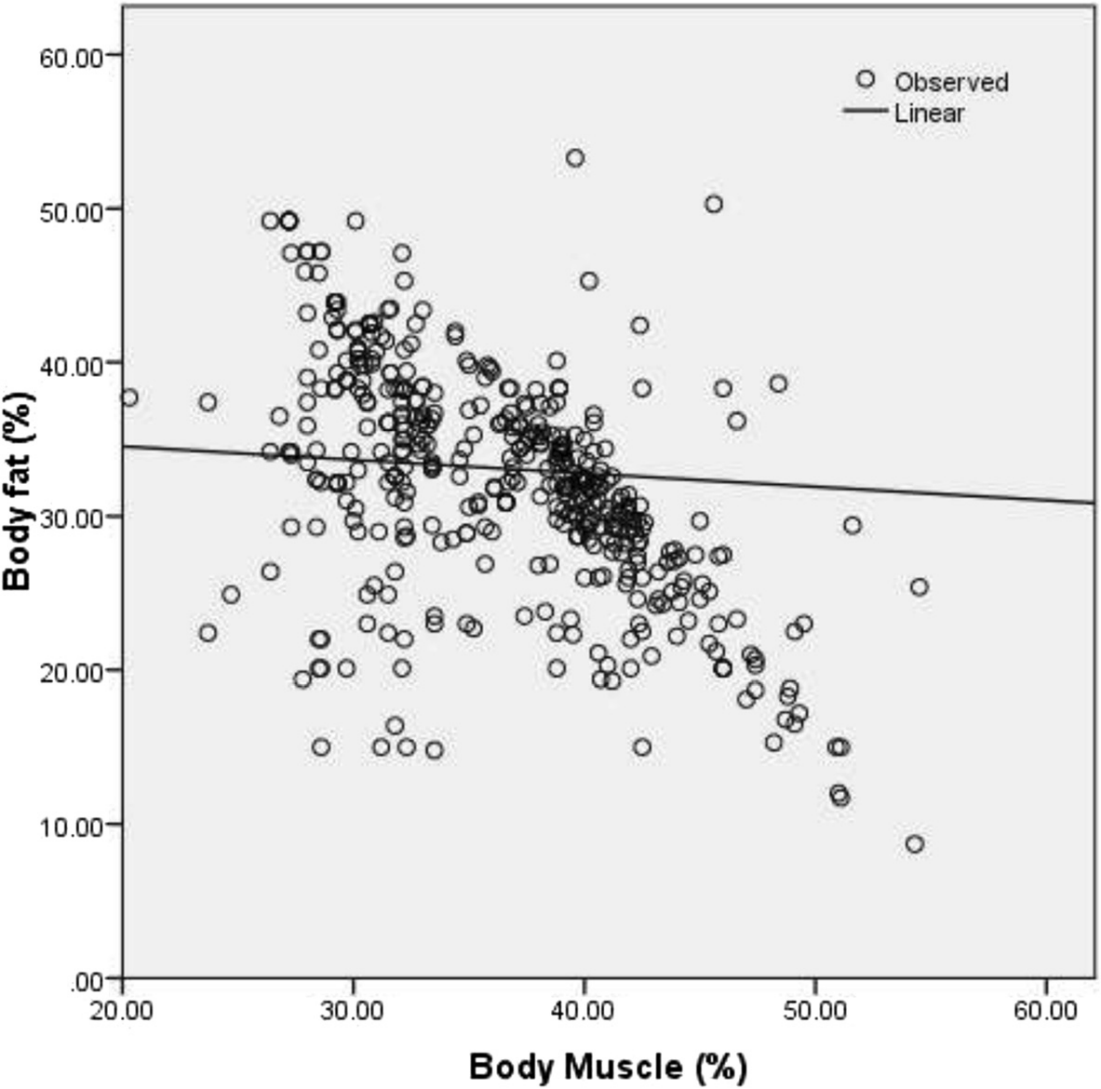
**Association of body muscle content with body fat percentage.** (P <0.001) (spearman correlation co-efficient r_s_ = −0.53).

**Figure 3:**
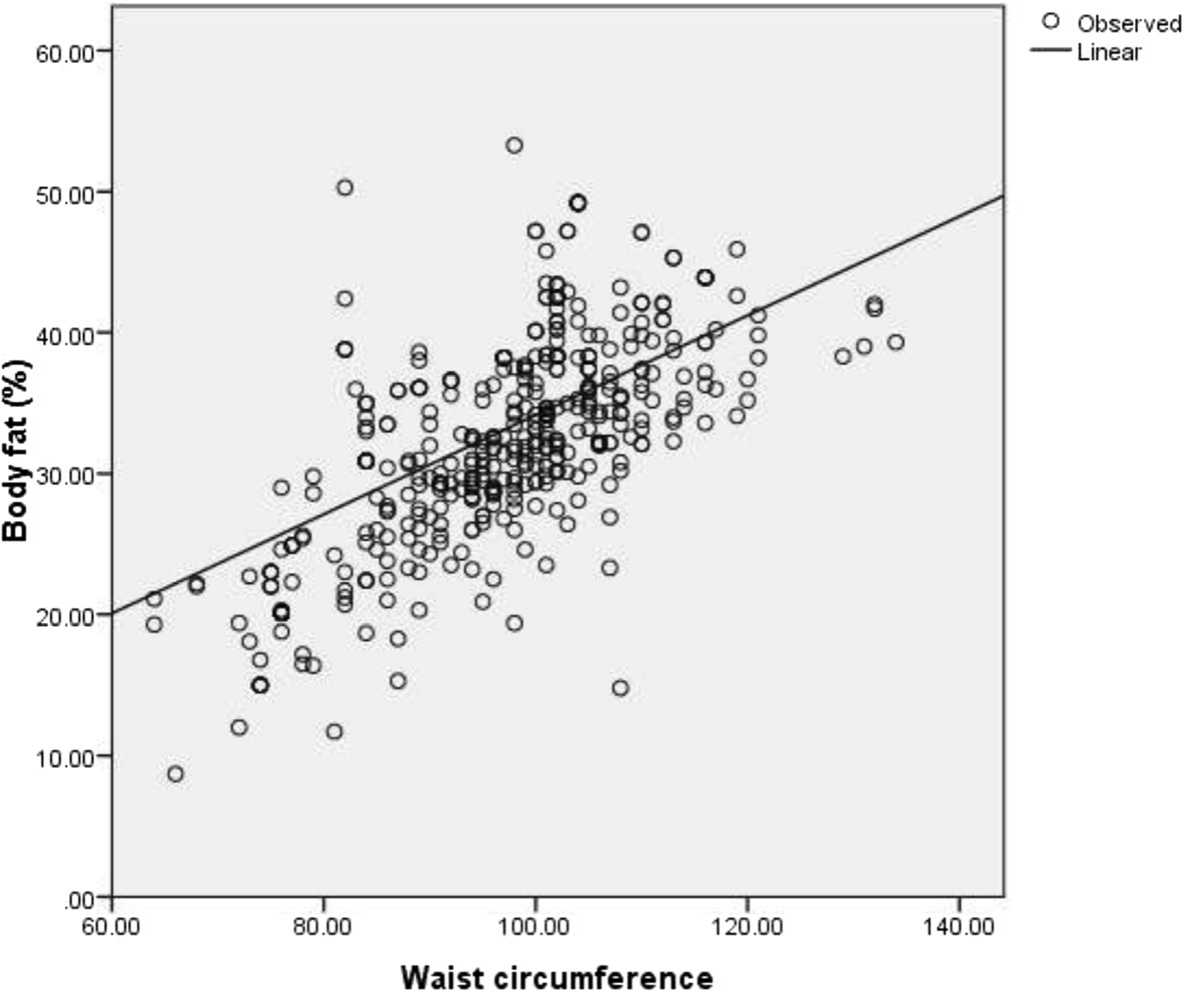
**Association of waist circumference with body fat percentage.** (P <0.001) (spearman correlation co-efficient r_s_ = 0.65).

**Figure 4:**
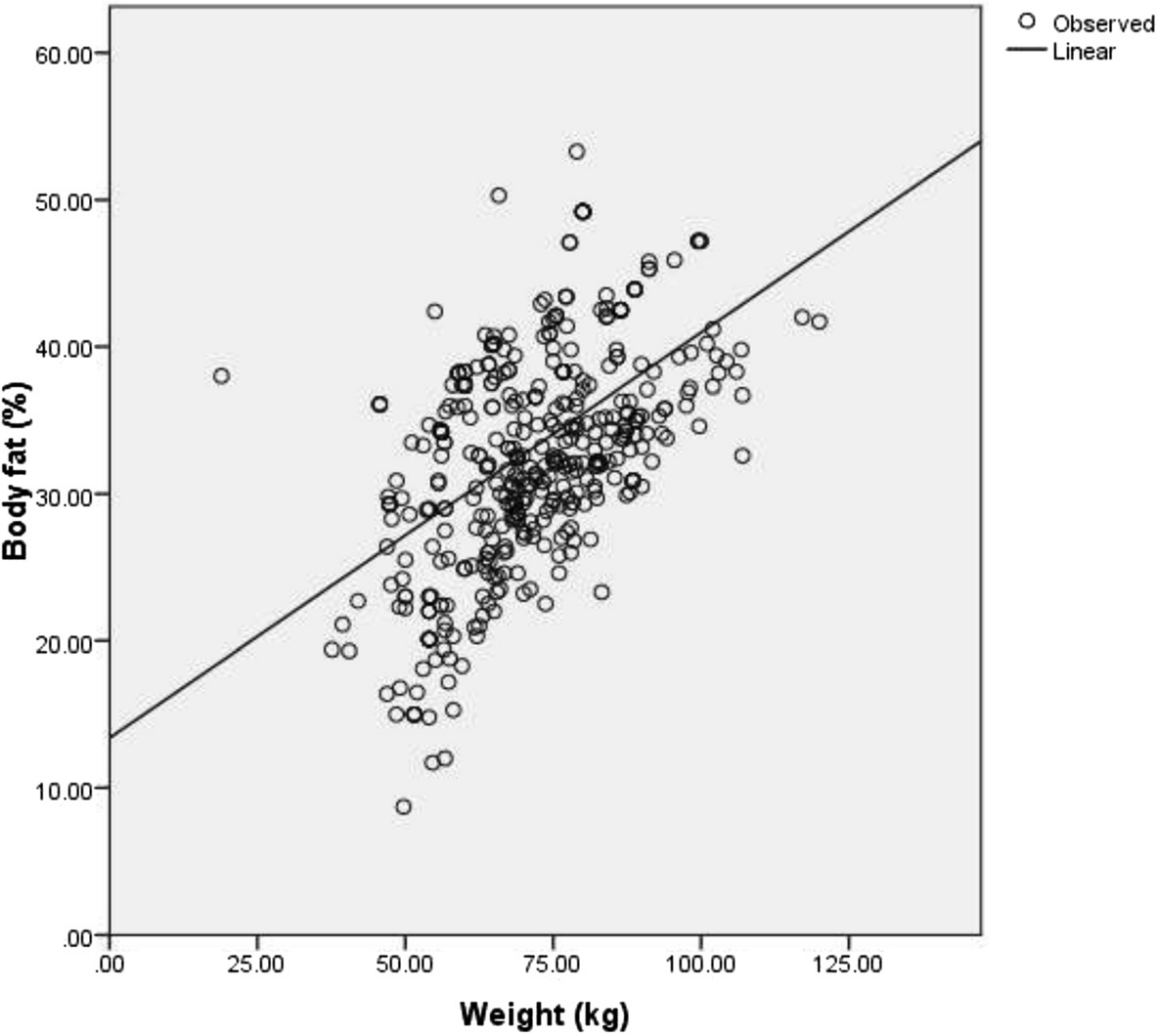
**Association of weight with body fat percentage.** (P <0.001), (spearman correlation co-efficient r_s_ =0.51).

**Figure 5:**
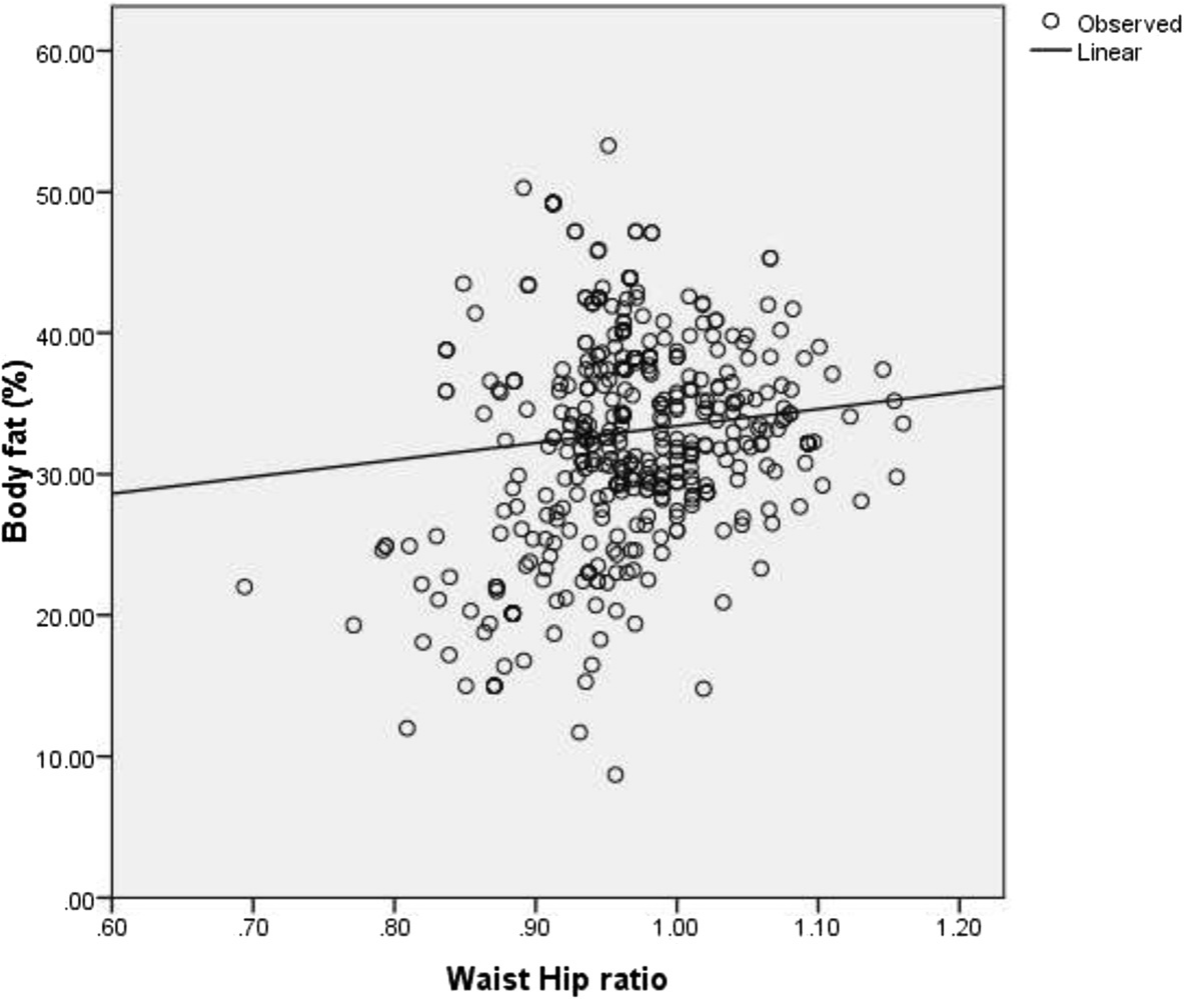
**Association of waist-hip ratio with body fat percentage.** (P <0.001 spearman correlation co-efficient r_s_ = 0.31).

**Figure 6:**
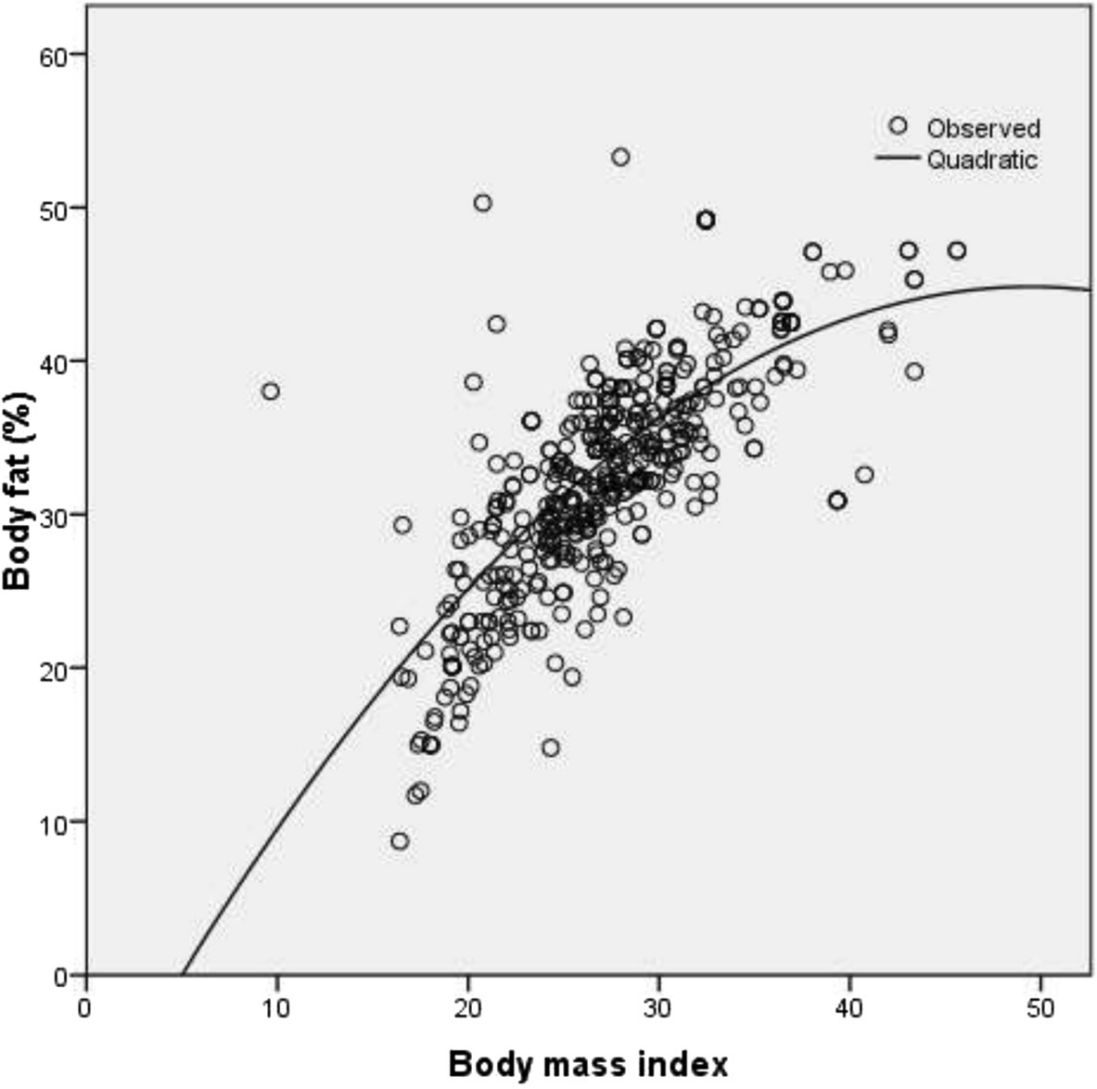
**Association of body mass index with body fat percentage.** P <0.001, spearman correlation co-efficient r_s_ = 0.74).

Total activity-METS (Figure 1) and muscle mass (Figure 2) had a significantly inverse linear association with BF% whereas; WC (Figure 3), weight (Figure 4) and WHR (Figure 5) had a positive linear association. The relationship between BMI and BF% was quadratic (Figure 6).

Applying multiple linear regression, body fat percentage, weight, diastolic blood pressure, body mass index and score on the diet questionnaire had a significantly positive linear association with waist circumference after adjusting for socio-demographic variables (age, gender, co-morbid illnesses, smoking/tobacco use and family history) with a co-efficient of determination (r^2^) equal to 0.66 (Table 3).

**Table 3:** **Linear regression model with waist circumference as the dependant variable (r** ^**2**^ **= 0.66)**

**Model**	**Beta**	**t**	**P value**	**CI**	
**Body fat (%)**	0.058	2.43	0.015	0.013	0.124
**Tobacco Use**	0.047	2.114	0.035	2.344	0.088
**Diastolic BP (mmHg)**	0.070	3.266	0.001	0.023	0.094
**Age**	0.114	5.354	<0.001	0.062	0.133
**Gender**	−0.198	−8.410	<0.001	−3.377	−5.433
**BMI (kg/m** ^**2**^ **)**	0.606	25.163	<0.001	1.184	1.384
**Score on diet questionnaire**	0.109	5.184	<0.001	0.149	0.067

Table 4 shows the linear regression model with body fat percentage as dependant variable (r^2^ = 0.59). Waist circumference, waist-hip ratio, systolic and diastolic blood pressure had a positive linear association adjusting for socio-demographic variables. Similarly, Table 5 shows the linear regression model with BMI as dependant variable (r^2^ = 0.708). BF%, muscle content and WC had a positive linear association with BMI.

**Table 4:** **Linear regression model with body fat percent as dependant variable (r** ^**2**^ **= 0.59)**

**Model**	**Beta**	**t**	**P value**	**CI**	
**Diastolic BP(mmHg)**	0.251	8.24	<0.001	0.137	0.223
**Systolic BP (mmHg)**	0.247	8.072	<0.001	0.122	0.074
**Waist circumference (cm)**	0.1111	3.672	<0.001	0.044	0.144
**Waist-hip ratio**	0.273	4.425	<0.001	0.343	0.892
**Gender**	−0.232	−8.519	0.006	−5.379	−3.365
**Age**	0.146	6.126	<0.001	0.072	0.139
**Tobacco use**	0.048	1.942	0.048	0.011	2.115
**Family history of hypertension**	0.063	2.583	0.01	2.07	0.283

**Table 5:** **Linear regression model with BMI as dependant variable (r** ^**2**^ **= 0.708)**

**Model**	**Beta**	**t**	**P value**	**CI**	
**Age**	0.063	3.174	0.002	0.041	0.010
**Gender**	−0.155	−7.347	<0.001	−2.055	−1.189
**Co-morbid illnesses**	0.060	3.086	0.002	0.131	0.589
**Family history of Diabetes**	0.044	2.141	0.032	0.916	0.040
**Family history of hypertension**	0.093	4.494	<0.001	0.548	1.396
**Body fat percent**	0.106	3.845	<0.001	0.023	0.070
**Waist circumference (cm)**	0.525	25.125	<0.001	0.229	0.268
**Muscle mass (%)**	0.132	5.248	<0.001	0.041	0.081

## Discussion

Non communicable diseases like obesity is posing a double burden of disease in developing nations like Pakistan, which is prevalent not only in urban but also in less privileged population. We found that around 50% of the participants were either overweight or obese according to their calculated BMI. The prevalence of central obesity was even more alarming (57%) especially among females. This is consistent with another study in the past [22]. We also observed a higher dietary intake among males (P <0.001), as well as a higher physical activity score (Activity-MET) (P 0.04). The waist-hip ratio was high among 65% of the participants, not surprisingly; males depicted this change more than females. This is in contrast with a previous study in Pakistan, which showed higher WC measurements for women but similar WHR for males and females [23]. Although, a study showed that in men, WC, rather than WHR is the anthropometric index that most uniformly predicts the distribution of adipose tissue in the abdominal region [24], but it has also been shown that WHR predicts vascular endothelial function in healthy overweight adults [25] and both WC and WHR are predictors of cardiovascular diseases [26] with a significant association of WHR with myocardial infarction risk worldwide [27]. These findings of our study are quite shocking considering that the population tested belonged to a suburban dwelling. Previous studies in Pakistan have shown a 28% prevalence of overweight/obesity in an urban population keeping a BMI cut-off of 25 kg/m^2^ but more than half the population enrolled were of 15–29 years of age in that study [28]. Other studies have shown up to 50% prevalence of overweight/ obesity and abnormal WC and WHR in up to 50% of the urban population [16,19]. In a National Health Survey in 1990–94, among the middle age group rural population, prevalence of obesity was found to be 11% for men and 19% for women and up to 40% in urban areas. Although this was two decades back, yet the prevalence of overweight and obesity in Pakistan is even currently underestimated, as a cut-off of 25 kg/m^2^ is being used for an abnormal BMI [14].

The prevalence of diabetes was up to 11% and hypertension was 17% in this study which was also comparable with other studies in urban areas of Pakistan, for example in 2009 a study in Karachi found a prevalence of hypertension of 9.4% in an urban population in Karachi and 12.1% in Punjab [29]. It is also known that world over, prevalence of diabetes in rural population has quintupled over last 25 years in low- and middle-income countries [30]. Another observation was that the random blood glucose was raised in 12% of the participants and systolic blood pressure was estimated to be 130 mmHg or more in a quarter of the population. This means that there may be patients with unidentified high blood glucose and blood pressure among the known cases as well, however, the follow up for these interesting findings was beyond the scope of our study.

The BMI and body fat% correlation observed was interesting and of clinical significance. Due to limited resources we did not use the gold standard which is dual energy x-ray absorptiometry (DEXA) for measuring body composition and we consider this as a limitation but BF% was measured using a bioelectrical impedance analysis (BIA) scale, which is also considered an effective tool [31] with a good agreement between BIA and DEXA, in measuring body fat% [32].

The systolic and diastolic BP, WC and WHR had a significant association with BF% in the multiple linear regression model. Similarly, in an Indian study, the relationship of BP and body fat revealed a high risk of hypertension in both males and females according to their fat-mass index [33]. In another study among USA population, both BMI and waist circumference were strongly correlated with body fat percentage, although in our study only waist circumference and not BMI predicted the body fat% model [34]. In another study from USA, it was shown that BMI and WC are more closely related to each other than with body fat percentage, although in men, WC correlated more with body fat percentage than BMI whereas, in females BMI correlated more with BF% [35]. In contrast, we observed in our study WC correlated with BF% in both males and females (P <0.001).

Although the activity-MET weakly correlated with body fat% in simple linear regression but was insignificant in the body fat% model whereas, higher score on the diet questionnaire was associated with an increase in waist circumference but not body fat (p >0.05).

Interestingly, muscle mass also positively correlated with BMI although it had a negative correlation with body fat% in simple linear regression. This means that BMI may not be a lone predictor of CVD risk, but it is rather a combination and interaction of other components such as body composition, WC and WHR. Although, BMI is still generally considered a marker of adiposity and increased CVD risk but there is enough evidence to support that measures of abdominal adiposity like WC and WHR, and not BMI, are associated with an increased risk of CVD mortality [36] . Yet, there are studies in certain subsets of populations such as in elderly Korean women, showing that BMI has a stronger correlation with BF% than with WC [37].

Moreover, we found a quadratic relationship between BMI and BF% influenced by gender and age, which is also consistent with other studies with a curvilinear association, with weak or no association of BF% at lower BMI values [38,39]. Thus, age and gender, especially in South-Asian population need to be taken into account when BMI is used to predict BF% or body composition [40].

Higher systolic and diastolic blood pressure were associated with body fat percentage and diastolic BP was positively correlated with WC in this study, which is consistent with other studies in our population. Increasing age was also associated with higher levels of BMI and body fat percentage [23,41].

Body fat percentage and muscle mass both depicted the linear regression model BMI, so a conclusion can be drawn that body mass index alone cannot predict body adiposity. Waist circumference and waist-hip ratio along with body mass index may better predict body adiposity, in settings where body fat percentage cannot be measured. The clinicians therefore, have to be vigilant in addressing CVD risk factors, as the high BMI alone does not necessarily mean increased adiposity and hence an increased risk. Yet, the prevalence of central obesity, overweight and obesity is on the rise and has to be addressed. Even though a relationship of high CVD risk with obesity has been suggested, along with the proposal to incorporate BF measurement for early identification of high risk individuals, further longitudinal population based studies are required that will provide valuable insight to verifying the classification discrepancies and determine a working classification/ develop a true population dependent cut-off value for both BMI and BF for a high risk population such as Pakistan.

## Conclusion

Considering South Asian cut-offs for body mass index and central obesity, this study showed a high prevalence of certain CVD risk factors among a sub-urban low income population in our region, which was comparable to the urban population. Considering the rising prevalence of non-communicable diseases, body fat percentage, waist circumference, waist-hip ratio and body mass index measurements together are convenient and feasible means of screening and identifying population at risk and hence addressing it through public awareness and early detection.

## Additional file

Additional file 1:
**Operational definitions.**

## Abbreviations

BP: Blood pressure
BF%: Body fat percent
BMI: Body mass index
CVD: Cardiovascular diseases
IPAQ: International physical activity questionnaire
MEDFICTS: Meats, eggs, dairy, fried foods, fat in baked goods, convenience foods, fats added at the table, and snacks
MET: Metabolic equivalent of task
WHO: World Health Organization
WC: Waist circumference
WHR: Waist-hip ratio
