# Supplementary material for: Prevalence of obesity and overweight, its clinical markers and associated factors in a high risk South-Asian population
Source: BMC Obes. 2015 Mar 18;2:16. doi: 10.1186/s40608-015-0044-6 (PMC4510896; doi:10.1186/s40608-015-0044-6)
Supplement: Additional file 1: — Operational definitions. [file 40608_2015_44_MOESM1_ESM.docx]

### **Operational definitions**:

**BMI [6, 11]**

BMI= Weight in kilograms divided by height in meters squared.

| Classification | BMI (kg/m^2^) |
| --- | --- |
| Underweight | <18.5 |
| Normal | 18.5-23 |
| Increased risk (overweight) | 23.1-27 |
| High risk (obese) | >27.5 |

**Waist circumference (WC) [6, 11]**

Circumference of waist was measured by a tape, taken midpoint between the lowest rib and the iliac crest after the patient exhales while standing without shoes, both feet touching, and arms hanging freely. Normal values are: less than 80 cm for women and < 90 cm for men.

**Hip circumference (HC) and waist-hip ratio (WHR)**

Hip circumference was taken by measuring at the widest part of the buttocks as recommended by WHO. The ratio was calculated by dividing the WC by HC and ≥.90 cm among males and ≥ 0.85 cm among females was considered abnormal.

**Blood pressure (BP):**

BP measured by a mercury sphygmomanometer as recommended by American Heart Association. It was measured by a trained nurse after at least 5 minutes of sitting, from the left arm.

**Body composition:**

Body composition (percentage of body fat and muscle mass) was measured by bioelectrical impedance at recruitment using bio-electrical impedance (BIA) scale (BG55 Beurer, Germany). Such scales have been validated and used in various populations including Pakistani population.

**Activity median METS-minutes**

Data collected with IPAQ was calculated as a continuous measure. Median MET-minutes were computed for walking (W), moderate-intensity activities (M), and vigorous-intensity activities (V) using the following formulas:

MET values and Formula for computation of Met-minutes:

Walking MET-minutes/week = 3.3 x walking minutes x walking days

Moderate MET-minutes/week = 4.0 x moderate-intensity activity minutes x moderate days

Vigorous MET-minutes/week = 8.0 x vigorous-intensity activity minutes x vigorous-intensity days

A combined total physical activity MET-min/week was computed as the sum of Walking + Moderate + Vigorous MET-min/week scores.

**Body Fat percent (BF%)**

Measured by bioelectrical impedance.

For males

| **Age (years)** | **Underweight (%)** | **Normal(%)** | **Overweight (%)** | **Significantly overweight(%)** |
| --- | --- | --- | --- | --- |
| **≤ 19** | <3 | 3-22 | 22.1-27 | ≥27.1 |
| **20-29** | <3 | 3-23 | 23.1-28 | ≥28.1 |
| **30-39** | <3 | 3-24 | 24.1-29 | ≥29.1 |
| **40-49** | <3 | 3-25 | 25.1-30 | ≥30.1 |
| **≥50** | <3 | 3-26 | 26.1-31 | ≥31.1 |

For females:

| **Age (years)** | **Underweight** | **Normal** | **Overweight** | **Significantly overweight** |
| --- | --- | --- | --- | --- |
| **≤ 19** | <12 | 12-27 | 27.1-32 | ≥32.1 |
| **20-29** | <12 | 12-28 | 28.1-33 | ≥33.1 |
| **30-39** | <12 | 12-29 | 29.1-34 | ≥34.1 |
| **40-49** | <12 | 12-30 | 30.1-35 | ≥35.1 |
| **≥50** | <12 | 12-31 | 31.1-36 | ≥36.1 |

**Muscle mass:**

For simplicity, high and very high categories were merged in one category:

| **Gender** | **Age** | **Low** | **Normal** | **High** |
| --- | --- | --- | --- | --- |
| **Female** | 18-39 | <24.3 | 24.3-30.3 | ≥30.4 |
|  | 40-59 | <24.1 | 24.1-30.1 | ≥30.2 |
|  | 60-80 | <23.9 | 23.9-29.9 | ≥30 |
| **Male** | 18-39 | <33.3 | 33.3-39.3 | ≥39.4 |
|  | 40-59 | <33.1 | 33.1-39.1 | ≥39.2 |
|  | 60-80 | <32.9 | 32.9-38.9 | ≥39 |
